# Effects of exercise training on intrahepatic lipid content in humans

**DOI:** 10.1007/s00125-016-4037-x

**Published:** 2016-07-08

**Authors:** Bram Brouwers, Matthijs K. C. Hesselink, Patrick Schrauwen, Vera B. Schrauwen-Hinderling

**Affiliations:** 1 NUTRIM School of Nutrition and Translational Research in Metabolism, Maastricht University Medical Center +, Maastricht, the Netherlands; 2 Department of Human Biology and Human Movement Sciences, Maastricht University Medical Center +, Maastricht, the Netherlands; 3 Department of Radiology, Maastricht University Medical Center +, P.O. Box 616, 6200 MD Maastricht, the Netherlands

**Keywords:** Exercise, Human, Insulin sensitivity and resistance, Lipid metabolism, Non-alcoholic fatty liver disease, Prediction and prevention of type 2 diabetes, Review

## Abstract

Non-alcoholic fatty liver (NAFL) is the most common liver disorder in western society. Various factors may play a role in determining hepatic fat content, such as delivery of lipids to the liver, de novo lipogenesis, hepatic lipid oxidation, secretion of intrahepatic lipids to the circulation or a combination of these. If delivery of lipids to the liver outweighs the sum of hepatic lipid oxidation and secretion, the intrahepatic lipid (IHL) content starts to increase and NAFL may develop. NAFL is closely related to obesity and insulin resistance and a fatty liver increases the vulnerability to type 2 diabetes development. Exercise training is a cornerstone in the treatment and prevention of type 2 diabetes. There is a large body of literature describing the beneficial metabolic consequences of exercise training on skeletal muscle metabolism. Recent studies have started to investigate the effects of exercise training on liver metabolism but data is still limited. Here, first, we briefly discuss the routes by which IHL content is modulated. Second, we review whether and how these contributing routes might be modulated by long-term exercise training. Third, we focus on the effects of acute exercise on IHL metabolism, since exercise also might affect hepatic metabolism in the physically active state. This will give insight into whether the effect of exercise training on IHL could be explained by the accumulated effect of acute bouts of exercise, or whether adaptations might occur only after long-term exercise training. The primary focus of this review will be on observations made in humans. Where human data is missing, data obtained from well-accepted animal models will be used.

## Intrahepatic lipid accumulation

**Fig. 1:**
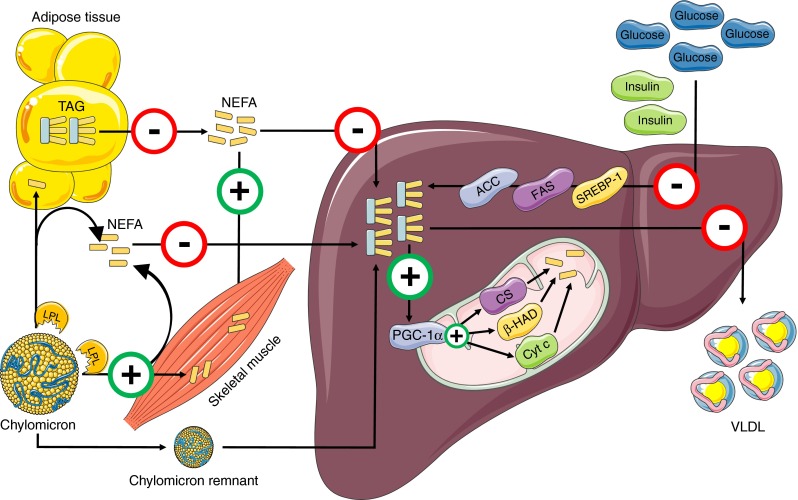
Pathways involved in hepatic lipid metabolism and the effect of exercise training on these pathways. Adipose tissue releases NEFA into the plasma via the process of lipolysis. Elevated fasting and postprandial plasma NEFA, originating from reduced inhibition of adipose tissue lipolysis, are taken up at the hepatic site. Fat originating from a meal is transported in chylomicrons. Adipose tissue and skeletal muscle take up fatty acids originating from the chylomicron–TAG pool via the action of LPL, while chylomicron remnants are taken up by the liver. However, when dietary fat availability is very high, the released fatty acids end up in the plasma NEFA pool via chylomicron–TAG ‘spillover’, and these NEFA can be taken up by the liver. Furthermore, hyperinsulinaemia increases hepatic glucose uptake, which activates DNL via sterol regulatory element binding protein-1 (SREBP-1), FAS and ACC. To compensate for the increased hepatic fat delivery and synthesis, hepatic TAG secretion via VLDL and hepatic TAG mitochondrial oxidation are upregulated. There is some evidence that exercise training decreases fasting and postprandial NEFA, most likely via a decrease in adipose tissue lipolysis. Moreover, exercise training increases uptake of NEFA by skeletal muscle and as a consequence lowers hepatic NEFA availability. Higher activity of LPL in skeletal muscle with exercise training increases the uptake of chylomicron–TAG by skeletal muscle, again lowering flux to the liver. In humans, exercise training lowers plasma insulin levels—a key player for activation of DNL—suggesting that exercise training might lower DNL activity, supported by animal data showing decreased ACC and FAS activity. Furthermore, animal data show that a decrease in IHL content with exercise training happens in the presence of increased PGC-1α and increased content of mitochondrial proteins used as markers of mitochondrial function (Cyt c, β-HAD and CS). Exercise training also lowers hepatic VLDL–TAG secretion, possibly as a consequence of lower hepatic TAG accumulation. Red circles represent inhibition of pathways with exercise training; green circles represent stimulation of pathways with exercise training

The prevalence of obesity has reached pandemic proportions [1]. Environmental factors such as consumption of high-energy diets and low levels of physical activity are likely to be underlying factors. Associated with obesity is the excessive storage of lipids in skeletal muscle, heart and liver, known as so-called ‘ectopic’ fat accumulation. In liver, this is diagnosed as non-alcoholic fatty liver (NAFL) if the fat accumulation occurs in the absence of high alcohol consumption. In obese people, the prevalence of NAFL may be as high as 50–70% [2, 3]. In a subset of patients, NAFL may develop into non-alcoholic steatohepatitis (NASH), cirrhosis or liver carcinoma [4]. Although NAFL is thought to be essentially benign and to be fully reversible, NAFL strongly correlates with hepatic and whole-body insulin resistance [2]. Insulin resistance is the earliest hallmark in the development of type 2 diabetes and the prevalence of NAFL in type 2 diabetes is estimated to be as high as 70% [2, 5]. Hepatic lipid accumulation can occur because of changes in the following: (1) NEFA plasma concentrations; (2) lipids originating from a meal; (3) de novo lipogenesis (DNL); (4) hepatic export of VLDLs or (5) hepatic fat oxidation (Fig. 1). These routes are briefly described below.

**Table:** 

**Conditions in which IHL accumulates**
• Increased hepatic NEFA uptake due to higher fasting and/or postprandial plasma NEFA concentrations
• Low partition of fatty acids to skeletal muscle (low chylomicron clearance by muscle and low NEFA uptake)
• Elevated DNL; activated by elevated plasma glucose and insulin concentrations
• Limited capacity to increase hepatic VLDL–TAG secretion and hepatic mitochondrial oxidation

### NEFA

Hepatic NEFA uptake is not strictly regulated [6]. Thus, higher plasma NEFA concentrations (predominantly originating from elevated adipose tissue lipolysis) directly relate to a higher hepatic NEFA influx, contributing to higher hepatic triacylglycerol (TAG) content [6, 7]. It has been shown that obese people with high intrahepatic lipid (IHL) content have a twofold higher rate of lipolysis than obese people with normal IHL content [8]. Using isotope tracers, Donnelly et al [9] elegantly showed that during fasting conditions 60% of the hepatic TAG content in patients with NAFL originated from plasma NEFA. Thus, high fasting NEFA concentrations associated with obesity might contribute to elevated IHL content [10]. However, not all studies found an association between fasting NEFA and IHL content [11–13], and some suggested that elevated postprandial NEFA concentrations (due to a diminished insulin-inhibitory effect on adipose tissue lipolysis) may be a more important factor contributing to ectopic lipid accumulation [14, 15]. Suppression of plasma NEFA was diminished during insulin infusion [11, 13] and during an OGTT [10] in individuals with elevated IHL content, when compared with obese [10, 13] and lean individuals [11] with normal IHL content. In addition, when comparing patients with NASH with healthy individuals, insulin-stimulated suppression of lipolysis was significantly impaired, while fasting lipolysis was comparable [16]. Thus, impaired insulin-stimulated suppression of lipolysis may be an underlying factor in NAFL.

### Lipids originating from a meal

IHL content can directly originate from dietary TAG, transported in chylomicron particles [17]. Release and uptake of TAG-containing chylomicrons occur via lipoprotein lipase (LPL), an enzyme located in the endothelium of adipose tissue and skeletal muscle [17, 18]. The chylomicron remnants then penetrate the fenestrations in the liver for receptor-mediated hepatic uptake [19]. Alternatively, dietary TAG can reach the liver via chylomicron–TAG ‘spillover’ into the serum NEFA pool after incomplete lipolysis via LPL in the adipose tissue and skeletal muscle [17, 19]. Various modulators of LPL, such as liver-produced apolipoproteins and angiopoietin-like proteins, are suggested to influence postprandial fat deposition in the liver [18]. In individuals with NAFL, approximately 15% of the hepatic TAG pool originates from dietary NEFA when a standard diet is consumed [9]. In isoenergetic conditions, the dietary fat concentration seems to be a determinant for the effect on IHL content [20–22]. More specifically, consuming an isoenergetic low-fat diet decreased IHL content [21, 22], while consuming an isoenergetic high-fat diet increased IHL content [21, 22]. Contrary to isoenergetic conditions, both low- and high-fat hypoenergetic diets decreased IHL content, often accompanied by a reduction in body weight [23]. In turn, high-carbohydrate and high-fat hyperenergetic diets markedly increased IHL content [23]. Carbohydrate overfeeding with fructose [24] or with glucose [24, 25] both significantly increased IHL content in lean healthy men [24] and overweight/obese men and women [25]. Thus, in energy imbalance, it appears that the composition of the diet may be less important, and that energy intake might be the main determinant for changes in IHL content.

### DNL

DNL was initially thought to account for only a small part of the hepatic TAG content in humans. However, insulin is a strong stimulator of DNL [26] and thus in people with hyperinsulinaemia (a common observation in NAFL) DNL may be increased. Indeed, DNL was 5.3- and 3.7-fold higher in hyperinsulinaemic obese individuals in comparison with more insulin-sensitive lean and obese individuals, respectively, after consumption of an isoenergetic high-fat, low-carbohydrate diet for 5 days [27]. In patients with NAFL, DNL contributed up to one-fourth of the hepatic TAG pool measured after an overnight fast [9], and DNL was significantly higher in insulin-resistant individuals with NAFL than in people with low IHL content [28]. Sevastianova et al [25] found that DNL increased in proportion to the increase in IHL content after 3 weeks of carbohydrate overfeeding, directly supporting a role for DNL in hepatic lipid accumulation.

### Hepatic VLDL metabolism

Rodents fed a methionine- and choline-deficient (MCD) diet, thereby compromising the assembly of VLDL, are commonly used to investigate NAFL [29]. However, decreased VLDL production is generally not thought to be an underlying factor in the development of NAFL in humans and has only been described in relation to rare apolipoprotein B-100 (ApoB-100) mutations [30]. Contrarily, elevated VLDL–TAG secretion rate upon higher IHL content has been observed repeatedly after an overnight fast [28, 31–33] and during the postprandial phase [34, 35]. It is, however, not yet completely clear how IHL content determines the VLDL–TAG secretion rate. While one study suggested a linear relation between IHL content and VLDL–TAG secretion [36], another study found a rise-to-plateau relationship [31]. The latter study described a linear relationship between IHL content and VLDL–TAG secretion in individuals with low IHL content, but not in those with NAFL. Interestingly, the latter study found that, while VLDL–TAG secretion was higher in people with NAFL, VLDL–ApoB-100 production was unchanged [31]. Thus, the liver did not increase VLDL particle production. Therefore, VLDL–TAG secretion might reach its maximum when maximal TAG load in the VLDL particles is reached, and hepatic TAG secretion cannot further compensate for increasing hepatic lipid delivery.

### Hepatic mitochondrial metabolism

Lipids are the preferred hepatic energy source during the fasted state [37] and the insulin-resistant fatty liver tends to activate hepatic oxidative metabolism [13, 38]. Hepatic mitochondrial function is elevated in individuals with NAFL, compared with obese controls, and positively correlates with IHL content [8]. In liver biopsies, Koliaki et al [39] found that mitochondrial respiration was higher in obese individuals with and without NAFL compared with lean control individuals, whereas mitochondrial content was comparable. Thus, NAFL develops in the presence of increased hepatic mitochondrial respiration, suggesting that increased lipid oxidation (and VLDL–TAG secretion) cannot fully compensate for the increased lipid delivery. Interestingly, mRNA expression of key components for mitochondrial biogenesis was markedly reduced in individuals with NAFL [39]. This could serve as a precedent for the decreased mitochondrial function observed in individuals with NASH [39–42]. Indeed, in NASH patients, activity of all mitochondrial respiratory chain complexes is diminished [42] and hepatic mitochondrial respiration is significantly impaired [39].

## Effects of exercise training on IHL content

### Liver fat metabolism

Exercise training is a recognised cornerstone in the treatment of obesity and its co-morbidities and recent studies have shown that exercise training might also have beneficial effects on IHL content [43–51]. Palmer and Schaffner [52] made one of the earliest observations of a possible link between regular physical activity and NAFL. These workers noted normalisation of alanine aminotransferase (ALAT) plasma levels in overweight patients with NAFL who had reduced their body weight by ≥10% via combined dietary restriction and regular unstructured physical activity. Since then, several studies have used proton magnetic resonance spectroscopy (^1^H-MRS), the non-invasive gold standard method with which to measure IHL content, to measure directly a reduction in IHL content with a combined weight loss and exercise training programme [53]. Furthermore, in the limited number of liver biopsy studies available, weight loss and exercise training have been found to significantly improve steatosis, lobular inflammation, ballooning injury and NAFLD activity score in patients with NASH [53]. Recently, there has been interest in whether exercise training per se, in the absence of weight loss, can reduce IHL content as well. Until now, however, only a few longitudinal studies that used ^1^H-MRS have investigated the direct effect of exercise training on IHL content in humans [43–51, 54] (Table 1).

**Table 1:** Main outcomes of studies that measured the effect of exercise training on IHL (measured with ^1^H-MRS) in humans

Study/participant type	Training protocol	Length of intervention	Intensity of exercise training protocol	Effect on IHL	Effect on other variables
van der Heijden et al (2010) [51]					
Obese Hispanic adolescents	Supervised AET (*n* = 15)	12 weeks	4×/week, 30 min; 70% of $\overset{.}{V}{\mathrm{O}}_{2\mathrm{peak}}$	↓	↓FM, ↓VAT, ↑IS, ↓f-Ins, =f-Glu
Lean Hispanic adolescents	Supervised AET (*n* = 14)	12 weeks	4×/week, 30 min; 70% of $\overset{.}{V}{\mathrm{O}}_{2\mathrm{peak}}$	No change	=FM, =IS, =f-Ins, =f-Glu
Sullivan et al (2012) [50]					
Obese NAFL patients	Unsupervised AET (*n* = 12)	16 weeks	5×/week, 30–60 min; 45–55% of $\overset{.}{V}{\mathrm{O}}_{2 \max }$	↓ (vs non-exercising control)	=BW, =FM, =f-NEFA
Pugh et al (2014) [49]					
Obese NAFL patients	Supervised AET (*n* = 13)	16 weeks	3×/week; 30 min at 30% of HRR (weeks 1–4), 30 min at 45% of HRR (weeks 5–8), 45 min at 45% of HRR (weeks 9–12), 45 min at 60% of HRR (weeks 13–16)	↓	↓f-Glu, =IS, =BW, =VAT, =f-Ins
Lee et al (2012) [47]					
Obese adolescent boys	Supervised AET *n* = 16)	3 months	3×/week; 40 min at 40% of $\overset{.}{V}{\mathrm{O}}_{2 \max }$ (week 1), 60 min at 60–75% of $\overset{.}{V}{\mathrm{O}}_{2 \max }$ (weeks 2–12)	↓ (vs non-exercising control)	↓BW, ↓FM, ↓VAT, =IS, =f-Glu, =f-Ins =2 h-Glu, =2 h-Ins
Obese adolescent boys	Supervised RET (*n* = 16)	3 months	3×/week, 60 min, 10 exercises, 2× 8–12 repetitions; 60% of 1RM (weeks 1–4), to fatigue (week 5–12)	↓ (vs non-exercising control)	↓BW, ↓FM, ↓VAT, ↑IS, =f-Glu, =f-Ins, =2 h-Glu, =2 h-Ins
Lee et al (2013) [48]					
Obese adolescent girls	Supervised AET (*n* = 16)	3 months	3×/week; 40 min at 40% of $\overset{.}{V}{\mathrm{O}}_{2 \max }$ (week 1), 60 min at 60–75% of $\overset{.}{V}{\mathrm{O}}_{2 \max }$ (weeks 2–12)	↓ (vs non-exercising control)	↓FM, ↓VAT, ↑IS, ↓f-Ins, =f-Glu, =2 h-Glu, =2 h-Ins
Obese adolescent girls	Supervised RET (*n* = 16)	3 months	3×/week, 60 min, 10 exercises, 2 × 8–12 repetitions; 60% of 1RM (weeks 1–4), to fatigue (weeks 5–12)	No change (vs non-exercising control)	↓FM, =VAT, =IS, =f-Glu, =f-Ins, =2 h-Glu, =2 h-Ins
Johnson et al (2009) [46]					
Obese men and women	Supervised AET (*n* = 19)	4 weeks	3×/week, 30–45 min; 50% (week 1), 60% (week 2), 70% (weeks 3 and 4) of $\overset{.}{V}{\mathrm{O}}_{2 \max }$	↓ (vs non-exercising control)	↓VAT, ↓f-NEFA, =BW, =f-Glu, =f-Ins
Hallsworth et al (2011) [45]					
Men and women with NAFL	Supervised RET (*n* = 11)	8 weeks	3×/week, 8 exercises, 45–60 min; 50% (weeks 1–6), 70% (weeks 7 and 8) of 1RM	↓	↑IS, ↓f-Glu, =BW, =FM, =VAT, =f-NEFA, =f-Ins
Finucane et al (2010) [44]					
Older men and women	Supervised AET (*n* = 50)	12 weeks	3×/week, 60 min; 50% (weeks 1–4), 60% (weeks 5–8), 70% (weeks 9–12) of W_max_	↓ (vs non-exercising control)	↓BW, ↓f-Ins, ↓2 h-Glu, ↓2 h-Ins, =FM, =f-Glu
Bacchi et al (2013) [43]					
Patients with type 2 diabetes and NAFL	Supervised AET (*n* = 14)	4 months	3×/week, 60 min; 60–65% HRR	↓	↓FM, ↓VAT, ↑IS
Patients with type 2 diabetes and NAFL	Supervised RET (*n* = 17)	4 months	3×/week, 9 exercises, 3 × 10 repetitions; 70–80% of 1RM	↓	↓FM, ↓VAT, ↑IS
Shojaee-Moradie et al (2007) [54]					
Overweight healthy men	Supervised AET (*n* = 10)	6 weeks	3×/week, 20 min; 60–85% of $\overset{.}{V}{\mathrm{O}}_{2 \max }$	No change	↑IS, ↓f-NEFA, ↓IS-NEFA, =BW, =FM

1RM, 1 repetition maximum; 2 h-Glu, 2 h glucose (OGTT); 2 h-Ins, 2 h insulin (OGTT); AET, aerobic exercise training programme; BW, body weight; f-Glu, fasting glucose; f-Ins, fasting insulin; FM, fat mass; f-NEFA, fasting NEFA; HRR, heart rate reserve; IS, insulin sensitivity; IS-NEFA, insulin-stimulated NEFA; RET, resistance exercise training programme; VAT, visceral adipose tissue; $\overset{.}{V}{\mathrm{O}}_{2 \max }$, maximal oxygen uptake; W_max_, maximal performance

In older individuals, 12 weeks of supervised aerobic exercise training for 1 h a day decreased IHL content significantly compared with a non-exercising control group [44]. Consistently, in obese, sedentary adults, 16 weeks of aerobic exercise training five times a week decreased IHL content by 10% in the absence of weight loss [50], and even 4 weeks of aerobic exercise training positively affected IHL content in a comparable group of individuals [46]. One study reported no change in IHL content in overweight healthy men after 6 weeks of aerobic exercise training [54], but exercise was limited to only three sessions of 20 min per week.

In addition, resistance exercise training for 8 weeks was found to reduce IHL content by 13% in obese, sedentary adults [45]. In the study of Bacchi et al [43], it was demonstrated that aerobic training and resistance training decreased IHL content to a similar extent (25–30% decrease from baseline). Van der Heijden et al [51] showed that 12 weeks of aerobic exercise training was also effective in lowering IHL content in younger people, reporting a decrease in IHL content from 8.9% to 5.6% in obese Hispanic adolescents. Interestingly, at least during adolescence, the sex of an individual seems to exert an effect. In the studies performed by Lee et al [47, 48], adolescent boys showed decreased IHL content after both aerobic and resistance exercise [47], while girls only benefited from aerobic exercise training [48].

While the finding that exercise training reduces IHL content is very reproducible, the mechanisms by which exercise training affects IHL content in humans are still unresolved. The simplest view is that the increase in energy expenditure due to physical activity simply induces a negative energy balance, which in turn may result in the mobilisation of hepatic lipids as a substrate to fuel the energy deficit. Most exercise training studies in which IHL content is decreased, however, do not show significant changes in body mass [45, 46, 50] or fat and fat free mass [44, 45, 50]. In fact, only Bacchi et al [43] concluded that there was a significant reduction in BMI and fat mass after exercise training compared with pre-exercise conditions. In Bacchi’s 4 month supervised aerobic or resistance exercise training programme, patients with type 2 diabetes and NAFL performed, three times a week, either 60 min of aerobic exercise training (60–65% of heart rate reserve) or resistance exercise training focusing on nine major muscle groups (70–80% of the 1 repetition maximum). Two months before onset of the training programme participants met with a nutritionist who provided encouragement to follow a healthy diet, and this might have affected the observations. Energy intake was measured by a 3 day food recall just before and at the end of the exercise training intervention. After exercise training, energy intake decreased by 323.8 kJ/day (77.4 kcal/day) in the aerobic training group and 384.5 kJ/day (91.9 kcal/day) in the resistance training group. Therefore, the decrease in energy intake throughout the exercise training programme might have been responsible for reduced BMI and fat mass, rather than the exercise training programme itself.

As outlined above, hepatic lipid accumulation originates due to changes in the delivery of lipids to the liver, changes in hepatic lipid oxidation, changes in the secretion of IHLs to the circulation or a combination of these factors. Since exercise training has the potential to lower IHL content, it may possibly do so by affecting one or more of the pathways involved (see Fig. 1). Below, we review the current knowledge on how exercise training affects the potential pathways involved in hepatic lipid accumulation in humans, supported by rodent data when no human data is available. By doing so, we reveal gaps in the current knowledge and highlight possibilities for future clinical research.

### NEFA

Endurance-trained individuals are characterised by low fasting plasma NEFA concentrations [55], suggesting that regular exercise may have an effect on adipose tissue lipid NEFA uptake and lipolysis. In line with this, a 6 month hypoenergetic diet in combination with exercise resulted in decreased in vitro basal adipocyte lipolysis in obese, postmenopausal women [56]. Shojaee-Moradie and colleagues [54] reported a decreased basal glycerol rate of appearance (R_a_) and palmitate R_a_ (indicative of a lower rate of lipolysis) 72 h after a 6 week exercise training programme in sedentary overweight men. However, this was not accompanied by a reduction in IHL content. Other studies did not find exercise training-induced effects on adipose tissue lipolysis. For example, using adipose microdialysis in 17 healthy elderly women, interstitial glycerol levels were found not to be affected after 12 weeks of exercise training [57]. Of note, studies that do report a decrease in adipose tissue lipolysis often observed a parallel decreases in (visceral) adipose tissue mass [54, 56], which can explain part of the observed effect on plasma NEFA concentrations.

Exercise training-induced decreases in IHL content do not necessarily occur in parallel with decreased plasma NEFA concentrations in the fasted state. Several studies report a decrease in IHL content even though fasted plasma NEFA concentrations remained unchanged [45, 50]. Exercise training also promotes whole-body insulin sensitivity and hence might also promote insulin-stimulated suppression of adipose tissue lipolysis [58]. We previously found that 12 weeks of combined aerobic and resistance exercise training improved insulin-mediated suppression of plasma NEFA in both individuals with type 2 diabetes and healthy controls matched for BMI [59]. Similarly, in obese men, 12 weeks of dynamic strength training did improve insulin-stimulated suppression of plasma NEFA [60]. Nevertheless, absolute NEFA levels during insulin infusion were comparable with pre-intervention concentrations, and the effect was mainly due to an increase in fasting plasma NEFA levels after the exercise training protocol. In contrast, no change in insulin-stimulated suppression of plasma NEFA was observed after 12 weeks of aerobic exercise training in people with impaired glucose tolerance [61, 62] and/or impaired fasting glucose [61]. In non-obese sedentary women insulin-stimulated plasma NEFA suppression improved after 9 months of high intensity (80% $\overset{.}{V}{\mathrm{O}}_{2 \max }$), but not after moderate-intensity (65% $\overset{.}{V}{\mathrm{O}}_{2 \max }$), aerobic exercise training [63].

Next to the potential effect on adipose tissue lipolysis, exercise training may also favour the clearance of plasma NEFA by skeletal muscle. In this respect, it is well established that endurance training improves whole-body fat oxidation [57, 64] and that this is accompanied by higher plasma NEFA uptake in skeletal muscle. With exercise training, this increased capacity for plasma NEFA uptake into the myocyte has been associated with an upregulation of membrane-associated plasma NEFA transport proteins [65]. Using 14(*R*,*S*)-[^18^F]6-thia-heptadecanoic acid (^18^F-FTHA, a plasma NEFA analogue that is transported across the cellular membrane but is not further metabolised) during a hyperinsulinaemic–euglycaemic clamp, it was found that, compared with healthy sedentary individuals, athletes possessed a higher plasma NEFA uptake in skeletal muscle, while hepatic retention of plasma NEFA was 20% lower [66]. Moreover, using the same plasma NEFA analogue in a monozygotic twin study, hepatic plasma NEFA uptake was 33% lower in the more active twin in comparison with the less active twin during a low-intensity 90 min knee extension exercise [67].

Taken together, exercise training may lower IHL content via effects on plasma NEFA. Whereas the effects on adipose tissue lipolysis are less clear, exercise training does seem to improve plasma NEFA uptake in skeletal muscle. This might lower the plasma NEFA availability for the liver, lowering hepatic plasma NEFA uptake and changing fatty acid partitioning from liver to skeletal muscle.

### Lipids originating from a meal

In addition to its effects on plasma NEFA levels, exercise training may also affect postprandial lipid metabolism. It is known that exercise training lowers the serum TAG response to a fatty meal [68] and the prevalence of postprandial hypertriacylglycerolaemia is significantly higher in sedentary people than in trained people [69]. The reduction in postprandial hypertriacylglycerolaemia associated with endurance exercise adaptations is due to a decrease in chylomicron–TAG half-life [70]. The difference in TAG half-life between athletes and sedentary men is most probably due to a direct effect of exercise training on LPL-mediated TAG removal to skeletal muscle [70].

Changes in the activity of LPL may play a major role in the more favourable lipoprotein–lipid profile of physically active individuals. Heparin is known to release LPL from its endothelial binding sites into the circulating blood [71–73] and post-heparin plasma LPL activity has been reported to be higher in endurance-trained individuals than in inactive controls [72, 73]. Furthermore, post-heparin LPL activity was reported to be increased after 20 weeks of endurance training in healthy, sedentary individuals [74]. The activity of LPL measured shortly after heparin infusion represents that of LPL derived from skeletal muscle rather than from adipose tissue [71]. This suggests that with exercise training postprandial TAGs are more likely to be taken up by the skeletal muscle.

Hepatic lipase (HL) plays an important role in lipoprotein remodelling [74], hydrolysing TAG and phospholipids from chylomicrons, HDL, intermediate-density lipoprotein (IDL) and LDL [75, 76], so that TAGs are taken up by the liver. It was shown that post-heparin HL activity reduced significantly after 20 weeks of endurance training [74], and this might result in lower postprandial hepatic TAG uptake. Although these indirect measurements suggest postprandial TAG channelling towards skeletal muscle and away from the liver with exercise training, the effect on postprandial hepatic fat storage has not been investigated directly.

### DNL

The effect of exercise training on DNL has not been investigated yet in humans. In animal models, however, a limited number of studies have been performed. In OLETF rats, exercise training reduced [77, 78] or prevented [79] hepatic fat storage concomitant with decreases in levels of acetyl-CoA carboxylase (ACC) and fatty acid synthase (FAS), two key enzymes in hepatic de novo fatty acid synthesis. Thus, at least in rats, there seems to be a link between exercise training-induced effects on IHL content and DNL activity. In all these animal studies, exercise training significantly reduced fasting glucose and fasting insulin levels [77–79]. Insulin is known to stimulate DNL from glucose [26], and lowered plasma concentrations of insulin and glucose may therefore blunt DNL. It is well documented that plasma concentrations of insulin and glucose are lower in endurance-trained individuals [80], due to improved whole-body insulin sensitivity [55]. This holds true for the fasted situation but even more so for the metabolic response to a meal. In exercise training studies wherein IHL content was lowered, significant decreases in fasting insulin [44, 48, 51], fasting glucose [45, 49], 2 h (OGTT) glucose [44] and 2 h (OGTT) insulin [44] upon exercise training have been reported. Taken together, while there is no direct human data on DNL activity, the observation of lower glucose and/or insulin levels in humans is in line with the observations made in animal models. Direct measurement of DNL activity before and after exercise training could bring further mechanistic understanding of the IHL content-lowering effect of exercise training in humans.

### Hepatic VLDL metabolism

Although VLDL metabolism is generally not thought to be an underlying factor in the development of NAFL in humans, exercise training could still influence IHL content by modifying VLDL metabolism. As yet, no study has investigated the effect of exercise training-induced changes in VLDL metabolism on IHL content in humans, but some studies have investigated how exercise training influences VLDL secretion and clearance. For example, 6 months of supervised exercise training resulted in a significant decrease in VLDL–ApoB-100 secretion rate in obese individuals with type 2 diabetes [81]. The VLDL–ApoB-100 catabolic rate, representing removal of VLDL particles from the vascular compartment by complete hydrolysis to IDL or by direct removal via the hepatic VLDL receptor [81], did not change. Moreover, the VLDL–TAG/ApoB-100 ratio was not altered, suggesting there was no change in TAG content per VLDL particle secreted. Since VLDL–ApoB-100 secretion rate decreased, plasma VLDL–TAG concentrations decreased. These findings were confirmed in a 2 month exercise training experiment with sedentary, non-obese young men [82]. Stable isotope-labelled 1,1,2,3,3-[^2^H_5_]glycerol tracer infusion in the post-absorptive phase revealed VLDL–TAG concentrations to be reduced by 28%, due to a 35% reduction in the hepatic VLDL–TAG secretion rate, whereas no differences in VLDL–TAG clearance could be observed. Thus, with exercise training, there seems to be a decrease in hepatic VLDL–TAG secretion. Although this indicates beneficial adaptation in liver metabolism, it cannot explain why exercise training lowers IHL content, since a lower VLDL–TAG secretion would theoretically lead to greater intrahepatic fat storage. Therefore, the reduced VLDL–TAG secretion rate is most likely a consequence of the reduced IHL content upon exercise training.

### Hepatic mitochondrial metabolism

The effect of exercise training on hepatic mitochondrial function has not yet been investigated in humans. In rodents, several indices of hepatic mitochondrial content and function were assessed in OLETF rats after a voluntary wheel running [77, 79] or an exercise training programme [78]. In these OLETF rats, exercise training reduced [77, 78] or prevented [79] hepatic fat storage concomitant with a decrease in key intermediates in hepatic fatty acid synthesis. Next to the effect on hepatic steatosis and DNL, these exercised OLETF rats exhibited increased complete palmitate oxidation to CO_2_ [77–79]. Moreover, hepatic citrate synthase (CS) activity [78, 79], β-hydroxyacyl-CoA dehydrogenase (β-HAD) activity [78, 79] and cytochrome c (Cyt c) protein content [77] were significantly upregulated. High CS and β-HAD activity indicates increased aerobic capacity and mitochondrial density, whereas elevated Cyt c protein content suggests enhanced final steps of oxidative phosphorylation. How exercise training might improve hepatic mitochondrial function is still unclear but the mechanism might involve enhanced mitochondrial biogenesis. A study in mice showed that hepatic Cyt c and cytochrome oxidase complex I protein content increased upon exercise training in wild-type mice [83] but the increase was absent in mice deficient in peroxisome proliferator-activated receptor γ coactivator 1-α (PGC-1α) [83]. This suggests that PGC-1α is required for the exercise training-induced adaptations of mitochondrial oxidative proteins in mouse liver. Using phosphorus magnetic resonance spectroscopy to measure hepatic mitochondrial function in relation to changes in IHL content with exercise training should be performed to see whether the observations made in rodents also hold true for humans.

**Table:** 

**Key effects of exercise training on pathways that influence IHL content**	
Plasma NEFA	• Increase in plasma NEFA uptake by skeletal muscle
	• Some evidence for decrease in fasting and/or postprandial plasma NEFA
Dietary TAG	• Increase in LPL-mediated TAG uptake by skeletal muscle
	• Decrease in HL-mediated TAG uptake by liver
DNL	• Decrease in plasma insulin, a key player for the activation of DNL
	• In diabetes, exercise can decrease plasma glucose and hence decrease DNL
	• Lower ACC and FAS protein content, indicative for decreased de novo lipolysis activity (rodent data)
VLDL metabolism	• Decrease in hepatic VLDL–ApoB-100 and VLDL–TAG secretion, possibly as a consequence of lower hepatic TAG accumulation
Mitochondrial oxidation	• Increase in hepatic CS, β-HAD and Cyt c, indicative for increases in hepatic mitochondrial content and oxidative phosphorylation (rodent data)

## Effects of acute exercise on IHL content

### Liver fat metabolism

An alternative way to explain the effects of exercise training on IHL content is by considering the accumulating effects of single acute bouts of exercise. However, hepatic fat content is not decreased after acute exercise; in fact, some studies report that acute exercise results in a slightly increased fat content [84, 85].

It has been shown that post-exercise splanchnic NEFA uptake in individuals with type 2 diabetes increases by 25% on average compared with the pre-exercise state [86]. Using stable isotope techniques it was calculated that approximately 50% of whole-body re-esterification immediately after physical exercise (1 h at 60% of $\overset{.}{V}{\mathrm{O}}_{2\mathrm{peak}}$) occurs in the splanchnic area [87]. This data indicates that splanchnic (i.e. hepatic) NEFA re-esterification might be an important factor immediately after exercise. So, while long-term exercise training results in lower NEFA levels and decreased IHL content, NEFA are increased after acute exercise and hepatic TAG content tends to increase after acute exercise in the fasted state.

### DNL

While it is not yet known how DNL is affected by long-term exercise training in humans, there are some data on the acute effect of exercise. Rabøl and colleagues [88] investigated the effect of a single exercise bout on DNL in insulin-resistant individuals performing 45 min of exercise or rest. After IHL content was determined, study participants were given a high-carbohydrate liquid meal (55 energy% carbohydrates) and deuterium-labelled water to measure DNL rates. They showed that DNL activity was significantly reduced in the exercise condition and this prevented the increase in IHL content that occurred during the resting condition, indicating that exercise positively influenced the rate of DNL after a subsequent meal. In this study, plasma glucose and plasma insulin levels were the same in the exercise and resting conditions but acute exercise induced a threefold increase in postprandial muscle glycogen synthesis. Thus, the single exercise bout redirected plasma glucose towards storage in skeletal muscle instead of liver, most likely via non-insulin-stimulated skeletal muscle glucose uptake by translocation of GLUT-4 [89, 90]. Therefore, eventually, the accumulated lowering effect of single bouts of exercise on DNL, by redirecting plasma glucose towards skeletal muscle, might contribute to the exercise training-induced decreases in IHL content.

### Hepatic VLDL metabolism

Exercise training reduces VLDL–TAG secretion and during acute exercise there may be a similar response. Two studies using VLDL–TAG and palmitate tracers measured VLDL secretion directly during, and after 90 min of, aerobic exercise at 50% of the $\overset{.}{V}{\mathrm{O}}_{2 \max }$ [91, 92]. These studies showed that in both healthy lean individuals and in overweight untrained men hepatic VLDL–TAG secretion and clearance were reduced during exercise and in the early recovery phase. Furthermore, because VLDL–TAG secretion and clearance were both reduced to a similar extent, plasma VLDL–TAG concentrations were not changed. Therefore it can be suggested that, in the early recovery phase, changes in VLDL–TAG secretion and/or clearance do not contribute to decreases in plasma TAG levels.

Plasma TAG levels remain relatively low for 24 h after an acute exercise bout [93], after which they return to baseline values. Although changes in VLDL–TAG metabolism do not contribute to the decrease in plasma TAG in the early recovery phase, it might be that they contribute to the sustained lower plasma TAG the day after a single exercise bout [93]. It has been shown that moderate-intensity exercise bouts lasting at least 2 h reduce plasma TAG concentrations by approximately 30% [94–96], whereas shorter bouts of similar exercise have no effect on plasma TAG concentrations [97, 98]. Consistently, it was found that 2 h of evening exercise (60% of the $\overset{.}{V}{\mathrm{O}}_{2\mathrm{peak}}$) did increase fasted VLDL-TAG clearance rate by approximately 40% on the following day, without affecting VLDL–TAG secretion in healthy, lean, young men [93]. In women, a comparable exercise protocol decreased VLDL–TAG secretion rate on the following day by approximately 22%, concomitant with increased VLDL–TAG clearance [99]. Thus, performing acute exercise for 2 h in the evening induces changes in VLDL–TAG metabolism on the following day and these changes positively affect TAG concentrations, mainly by causing increased clearance of VLDL–TAG from the plasma and only marginally involving changes in hepatic VLDL–TAG secretion.

## Conclusions

NAFL develops due to higher hepatic fat availability and synthesis, which is not fully compensated by increased secretion and oxidation of hepatic TAG. Weight loss plus exercise training is very effective in decreasing IHL content. When body weight is kept constant, exercise training by itself also brings about a distinct, but more modest, decrease in IHL content. Exercise training lowers IHL content most likely via a reduction in hepatic fat availability and synthesis, and an increase in hepatic TAG oxidation. A single bout of exercise seems to increase rather than decrease IHL content and elevated plasma NEFA during exercise and in the post-exercise period might be the main determinant. Clearly, further research needs to be performed to better understand the underlying pathways and systems involved in the beneficial effects of physical activity on hepatic metabolism and crosstalk with whole-body metabolism.

## Abbreviations

ACC: Acetyl-CoA carboxylase
ALAT: Alanine aminotransferase
ApoB-100: Apolipoprotein-B100
CS: Citrate synthase
Cyt c: Cytochrome c
DNL: de novo lipogenesis
FAS: Fatty acid synthase
^18^F-FTHA: 14(*R*,*S*)-[^18^F]6-thia-heptadecanoic acid
β-HAD: β-Hydroxyacyl-CoA dehydrogenase
HL: Hepatic lipase
^1^H-MRS: Proton magnetic resonance spectroscopy
IDL: Intermediate-density lipoprotein
IHL: Intrahepatic lipid
LPL: Lipoprotein lipase
MCD: Methionine- and choline-deficient
NAFL: Non-alcoholic fatty liver
NASH: Non-alcoholic steatohepatitis
PGC-1α: Peroxisome proliferator-activated receptor γ coactivator 1-α
TAG: Triacylglycerol
